# Rat Bone Marrow Mesenchymal Stem Cells-Derived Exosomes Promote the Proliferation, Invasion, and Metastasis and Inhibit Apoptosis of Colorectal Cancer Stem Cells

**DOI:** 10.1155/sci/7889271

**Published:** 2025-10-17

**Authors:** Yu Jing, Nan Yang, Shutao Yu, Huiran Qi, Tianqing Yu, Xingyu Chen, Shuyue Wei, Weiyan Zou, Junbin Wang

**Affiliations:** ^1^Department of Histology and Embryology, Bengbu Medical University, Bengbu 233030, Anhui Province, China; ^2^Department of Oncology, the First Affiliated Hospital of Bengbu Medical University, Bengbu 233004, Anhui Province, China; ^3^Clinical Medical College, Bengbu Medical University, Bengbu 233030, Anhui Province, China

**Keywords:** cancer stem cell, colorectal cancer, epithelial–mesenchymal transition, exosome, mesenchymal stem cell

## Abstract

**Objective:**

To evaluate the impact of exosomes derived from rat bone marrow mesenchymal stem cells (BM-MSCs) on the malignant properties of human colorectal cancer stem cells (CRC-CSCs) and the underlying mechanism involving epithelial–mesenchymal transition (EMT).

**Methods:**

Exosomes were isolated and characterized from rat BM-MSCs. Human CRC-CSCs were enriched from HCT116 cells and subsequently treated with the exosomes. Cellular functions, including proliferation, apoptosis, cell cycle progression, migration, and invasion, were assessed using cell counting kit-8 (CCK-8), colony formation, flow cytometry, and Transwell assays, respectively. In vivo tumorigenicity and lung metastasis were evaluated using a xenograft mouse model. Expression levels of EMT markers (E-cadherin, N-cadherin, and Vimentin) were analyzed by western blot, qPCR, and immunofluorescence.

**Results:**

BM-MSCs-derived exosomes were efficiently internalized by HCT116-CSCs. In vitro, exosome treatment significantly enhanced cell proliferation, migration, invasion, and cell cycle progression, while suppressing apoptosis. In vivo, exosomes promoted tumor growth and lung metastasis. Mechanistically, exosome exposure induced EMT, as evidenced by decreased E-cadherin expression and increased expression of N-cadherin and vimentin in both in vitro and in vivo models.

**Conclusion:**

Exosomes derived from rat BM-MSCs enhance the malignant phenotype and suppress apoptosis in human CRC-CSCs through the activation of the EMT pathway. These findings underscore the potential role of BM-MSC-derived exosomes in tumor microenvironment (TME) regulation and highlight their relevance as a potential therapeutic target.

## 1. Introduction

Colorectal cancer (CRC) remains a significant global health burden due to its high rates of metastasis and recurrence [1, 2]. A key contributor to this malignant progression is a distinct subpopulation of cells known as cancer stem cells (CSCs), which exhibit self-renewal capabilities, resistance to conventional therapies, and are primarily responsible for tumor initiation and dissemination [3–6]. Consequently, targeting CSCs has emerged as a promising strategy for improving CRC treatment outcomes [7–9].

The behavior of CSCs is not autonomous but is critically influenced by their surrounding tumor microenvironment (TME) [10, 11]. Within the TME, mesenchymal stem cells (MSCs), particularly those originating from bone marrow (BM-MSCs), are recruited to tumor sites and actively contribute to the regulation of tumor growth and metastasis [12, 13]. Our previous studies have shown that rat BM-MSCs directly enhance the invasive and migratory abilities of colorectal CSCs [14, 15]. However, the specific mechanisms underlying this tumor-promoting interaction remain poorly understood.

An important mechanism of intercellular communication within the TME is mediated by exosomes—nanoscale extracellular vesicles that transport bioactive molecules between cells [16, 17]. BM-MSCs are known to secrete exosomes that can influence the behavior of recipient cells [18]. Although MSC-derived exosomes have demonstrated therapeutic potential in certain contexts [19, 20], their role in modulating the malignant characteristics of colorectal cancer stem cells (CRC-CSCs) remains unclear.

Based on our previous findings, we hypothesized that the tumor-promoting effects of BM-MSCs on colorectal CSCs are primarily mediated through their secreted exosomes. Therefore, this study was designed to evaluate the impact of exosomes derived from rat BM-MSCs on the proliferation, apoptosis, invasion, and metastasis of human colorectal CSCs. Additionally, we aimed to investigate whether the epithelial–mesenchymal transition (EMT) process serves as a potential underlying mechanism.

## 2. Materials and Methods

### 2.1. Animals

Male Sprague–Dawley (SD) rats aged 3–4 weeks, weighing 80–100 g, and specific pathogen-free (SPF)-grade male BALB/c nude mice aged 4–6 weeks were purchased from Hangzhou Ziyuan Experimental Animal Technology Co., Ltd. (Hangzhou, China). All animals were housed in an SPF facility at the Experimental Animal Centre of Bengbu Medical University (Bengbu, China) and had free access to water and food.

### 2.2. Cell Culture and HCT116-CSCs Preparation

The microspheres of HCT116-CSCs were cultured and passaged as described previously [15]. Briefly, human CRC HCT116 cells (Cell Bank of the Chinese Academy of Sciences; Shanghai, China) were cultured with McCoy's 5A medium containing 10% fetal bovine serum (FBS) at 37°C with 5% CO_2_ until the cell confluence reached 80% to 90%. Cells were digested, prepared as single-cell suspensions at a density of 1 × 10^4^ cells/mL, and incubated in ultralow attachment culture dishes. The medium was with 1 mL of fresh medium every 2–3 days, and spheres were passaged every 5–8 days. Finally, cells from the third passage were harvested and divided into three groups, including the control group (no treatment), the Exo group (cocultured with 200 μg BM-MSCs-derived exosomes, this concentration was based on preliminary experiments and previous literature [14]), and the PBS group (treated with an equal volume of PBS). Cells were then cultured for a further 48 h and harvested for the subsequent experiments.

### 2.3. Isolation and Identification of BM-MSCs-Derived Exosomes

Rat BM-MSCs were selected for this study due to their accessibility, high exosome production capacity, and well-established isolation protocols in our laboratory, which facilitated the initial mechanistic investigation. BM-MSCs were isolated from 3–4-week-old SD rats. Briefly, rats were sacrificed by dislocation of the cervical vertebrae. The femurs and tibiae were aseptically removed, and the bone ends were excised. The bone marrow cavity was flushed with low-glucose Dulbecco's Modified Eagle's Medium (L-DMEM; GIBCO, Rockville, MD, USA) using a syringe. The flushed cell suspension was plated at a density of 1 × 10^6^ to 1 × 10^8^ cells/mL in 100 mm culture dishes and incubated at 37°C in a 5% CO_2_ incubator for 48 h. Upon reaching 80%–90% confluence, cells were passaged at a 1:2 ratio.

Log-phase BM-MSCs from passages 3–5 were used for exosome isolation. When cells reached 60%–70% confluence, the supernatant was discarded, and the cells was washed twice with PBS. The culture medium was then replaced with exosome-depleted FBS complete medium and cultured for another 48 h. The conditioned medium was collected and sequentially centrifuged at 300 × *g* for 10 min and 2000 × *g* for 15 min at 4°C to remove cells and debris. Exosomes were extracted and purified using the exosome extraction kit (Umibio Science and Technology Group; Shanghai, China) according to the manufacturer's instructions. Exosome concentration was measured using a bicinchoninic acid (BCA) assay, adjusted to 1 mg/mL, and stored at −80°C. Exosome morphology was observed by transmission electron microscopy, and particle size distribution was analyzed using Flow NanoAnalyzer. In addition, the expression of exosome-specific markers CD9 and CD63 was confirmed by western blot.

### 2.4. Exosome Uptake Assay

BM-MSCs-derived exosomes stored at −80°C were thawed on ice. For labeling, 7.5 μL PKH67 dye was diluted in 75 μL Diluent C and mixed with 300 μg of exosomes. The mixture was incubated for 1 min and then stood for 10 min. To remove excess dye, The mixture was diluted with 10 mL PBS and ultracentrifuged at 10,000 *g* for 60 min at 4°C. The pellet was washed twice with PBS and resuspended in PBS. HCT116-CSCs were then treated with 50 μg of PKH67-labeled exosomes (final concentration 10 μg/mL) and incubated. Exosome uptake was observed under a fluorescence microscope at 12, 24, and 48 h.

### 2.5. Western Blot Analysis

Cells were lysed using RIPA lysis buffer containing PMSF and a protease inhibitor cocktail (Beijing Solarbio Science & Technology Co., Ltd., Beijing, China) in a ratio of 100:1:0.15. Protein concentrations were determined using the BCA method. Equal amounts of proteins (30 μg) were separated with 10% SDS-PAGE and transferred onto 0.45 μm PVDF membranes. The membranes were blocked with 5% skim milk for 2 h at room temperature, incubated with the primary antibodies (1:2,000 dilution) overnight at 4°C, washed with TBST, and then incubated with the secondary antibodies (1:4,000 dilution) for 1 h at room temperature. After washing, protein bands were visualized using an ECL chemiluminescent substrate kit and imaged with a Bio-Rad gel imaging system.

### 2.6. Colony Formation Assay

Single-cell suspensions were prepared and seeded into 6-well plates at a density of 200 cells/well, with three replicate wells per group. Cells were cultured in McCoy's 5A medium, and the medium was changed every 3–4 days. After 2 weeks, the plates were removed, and the medium was discarded. Cells were washed twice in PBS, fixed with 4% paraformaldehyde for 20 min, and stained with crystalline violet for 20 min. After washing, colonies were observed, counted, and photographed under a microscope.

### 2.7. Cell Counting Kit-8 (CCK-8) Assay

Single-cell suspensions were prepared, and the cell density was adjusted to 3 × 10^4^ cells/mL. Then, 100 μL of cell suspension was added to each well of a 96-well plate and (4–5 replicate wells per group). The plate was incubated at 37°C, and at 0, 24, 48, and 72 h, 10 μL of CCK-8 solution was added to each well and incubated for another 2 h. The optical density (OD) was measured at 450 nm using a microplate reader, and a cell growth curve was plotted.

### 2.8. Apoptotic Assay

Single-cell suspensions were prepared and centrifuged at 1000 rpm for 5 min at 4°C. The pellet was washed twice with PBS and resuspended in 200 μL of binding buffer. Then, 4 μL of Annexin V-FITC was added, and the mixture was gently vortexed and incubated for 10 min at room temperature. After centrifugation, the pellet was resuspended in 190 μL of binding buffer and 4 μL of propidium iodide (PI) staining solution and incubated on ice. Apoptosis was analyzed by flow cytometry.

### 2.9. Transwell Migration and Invasion Assay

For the invasion assay, Matrigel was diluted to 250 μg/mL and applied to the upper chamber of a Transwell insert. The plate was incubated at 37°C for 1 h to allow the gel to solidify. For both migration (no Matrigel) and invasion (with Matrigel) assays, single-cell suspensions were prepared in serum-free medium at a density of 2 × 10^5^ cells/mL. Then, 200 μL of cell suspensions was added to the upper chamber, and 700 μL of complete medium containing 15% FBS was added to the lower chamber. After incubation for 48 h(migration) or 24 h (invasion), the chambers were removed. Cells on the upper surface were wiped away, and cells that had migrated or invaded to the lower surface were fixed with 4% paraformaldehyde, stained with 0.1% crystal violet, and counted under a microscope (five random fields per chamber). Experiments were performed in triplicate.

### 2.10. In Vivo Tumorigenicity Assay

Ten 4–6-week-old BALB/c nude mice were randomly divided into 2 groups (*n* = 5 per group), including the control group (injected with HCT116-CSCs pretreated with 200 μL PBS for 48 h) and the experimental group (injected with HCT116-CSCs pretreated with 200 μL BM-MSC-derived exosomes for 48 h). Cells were digested into single-cell suspensions at a density of 2.5 × 10^6^ cells/mL. Then, 100 μL of cell suspension was injected subcutaneously into the left inguinal region of each mouse. Six weeks postinjection, all mice were euthanized by cervical dislocation. Tumors and lungs were excised. Tumors were weighed and subjected to H&E and immunohistochemical staining. Lung tissues were stained with H&E to observe metastases. Immunohistochemistry was performed to determine the expression of E-cadherin (1:1000; GeneTex), N-cadherin (1:1000; GeneTex), and Vimentin (1:1000; GeneTex) in tumor tissues.

### 2.11. qPCR Assay

Total RNA was extracted from cells or tissues using TRIzol reagent (Invitrogen; Carlsbad, CA, USA) according to the manufacturer's instructions, transcribed into cDNA with the reverse transcription kit. qPCR was performed using specific primers (Table 1) under the following conditions: 95°C for 20 s, followed by 45 cycles at 95°C for 3 s and 56°C for 30 s. All assays were repeated in triplicate, and relative mRNA expression was quantified using the 2^−ΔΔCt^ method.

### 2.12. Immunofluorescence Staining

Single-cell suspensions were inoculated into ultra-low attachment 6-well plates for 48 h. Cells were harvested and seeded onto poly-L-lysine-coated coverslips and cultured for another 72 h. Cells were washed with PBS, fixed with 4% paraformaldehyde for 20 min, permeabilized with 0.5% Triton X-100 for 20 min, and blocked with goat serum for 30 min. Subsequently, cells were incubated with primary antibodies against E-cadherin, Vimentin, and N-cadherin (Abcam, 1:200) overnight at 4°C. After washing, cells were incubated with fluorescent secondary antibody (1:1,000) for 1 h at room temperature and counterstained with DAPI (1:50) for 5 min. Images were captured using a fluorescence microscope.

### 2.13. Statistical Analysis

Data were analyzed using SPSS 21.0 software (IBM Corporation; Armonk, NY, USA). Normally distributed measurement data were expressed as mean ± standard deviation. Differences between two groups were analyzed using an unpaired *t*-test, and comparisons among multiple groups were performed using one-way analysis of variance (ANOVA). A *p*-value < 0.05 was considered statistically significant.

## 3. Results

### 3.1. Isolation, Identification, and Uptake of Rat BM-MSCs-Derived Exosomes

**Figure 1:**
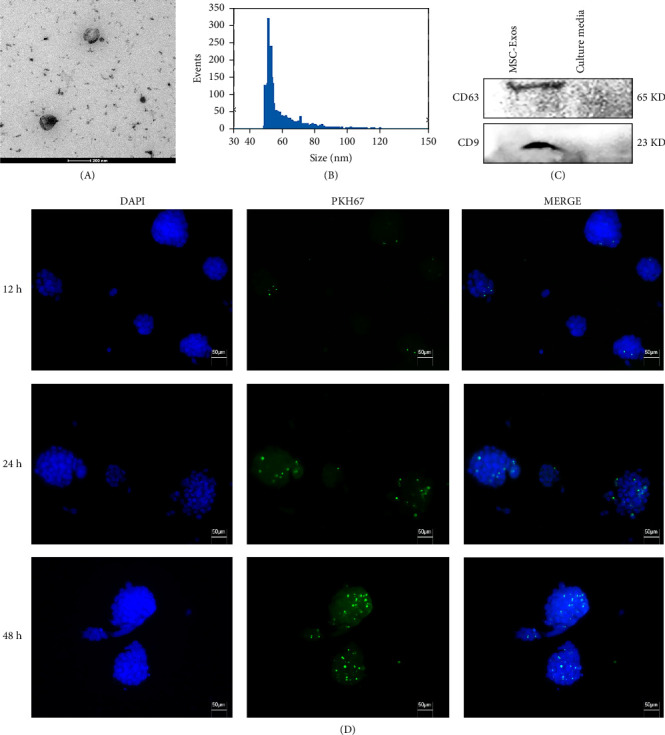
Characterization of rat bone-marrow mesenchymal stem cells-derived exosomes (BM-MSC-Exos). (A) Transmission electron microscopy of rat BM-MSC-Exos. Scale bar = 200 nm. (B) Flow nanoAnalyzer showing the size distributions of rat BM-MSC-Exos. (C) Western blot analysis of exosomal markers CD9 and CD63. Lane 1: rat BM-MSC-Exos; Lane 2: BM-MSCs culture medium. (D) Uptake of PKH67-labeled BM-MSC-Exos (green) by HCT116-CSCs over time. Nuclei were counterstained with DAPI (blue). Scale bar = 50 μm.

Transmission electron microscopy showed that the isolated BM-MSCs-derived exosomes exhibited a typical cup-shaped or spherical morphology with intact membranes (Figure 1A). Flow nanoAnalyzer confirmed a size distribution predominantly within the 30–200 nm range (Figure 1B). Western blot analysis demonstrated positive expression of the exosome-specific surface markers CD63 and CD9 (Figure 1C), confirming successful isolation. To track exosome uptake, PKH67-labeled exosomes were cocultured with HCT116-CSCs. Fluorescence microscopy showed the exosomes were internalized by HCT116-CSCs in a time-dependent manner, as indicated by the increasing number of green fluorescent granules within the spheres over 12, 24, and 48 h (Figure 1D).

### 3.2. BM-MSCs-Derived Exosomes Promote the Proliferation of HCT116-CSCs

**Figure 2:**
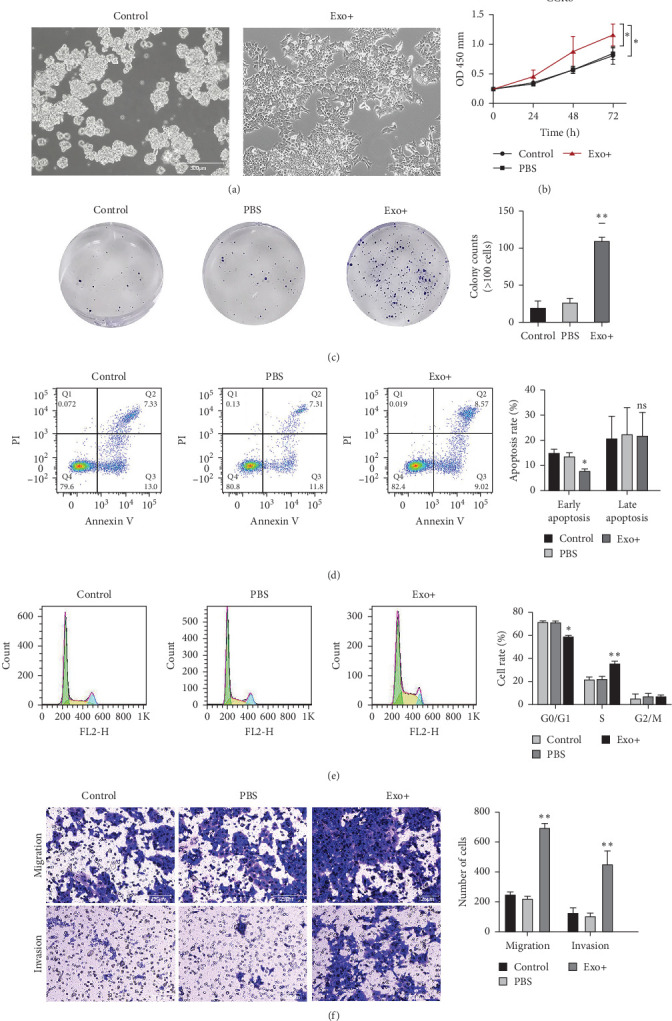
Effect of rat BM-MSC-Exos on biological behaviors of HCT116-CSCs. (A) Morphological changes of HCT116-CSCs after treatment with BM-MSC-Exos. Scale bar = 300 μm. (B) CCK-8 assay for cell viability. *⁣*^*∗*^*p*  < 0.05 vs. the PBS or control group. (C) Colony formation assay. *⁣*^*∗∗*^*p*  < 0.01 vs. the PBS or control group. (D) Flow cytometry for apoptosis analysis. *⁣*^*∗*^*p*  < 0.05 vs. the PBS or control group; ns, not significant. (E) Flow cytometry for cell cycle analysis. *⁣*^*∗*^*p*  < 0.05 vs. the PBS or control group; *⁣*^*∗∗*^*p*  < 0.01 vs. the PBS or control group. (F) Transwell migration and invasion assays. *⁣*^*∗∗*^*p*  < 0.01 vs. the PBS or control group. Scale bar = 125 μm.

Treatment with BM-MSC-derived exosomes induced morphological changes in HCT116-CSCs (Figure 2A). The CCK-8 assay showed the OD values in the Exo group were significantly higher than those in the PBS group and control group at 48 and 72 h, indicating enhanced cell viability (Figure 2B). Consistent with this, the colony formation assay revealed a significantly greater number of colonies in the Exo group compared to the control groups (Figure 2C), indicating that BM-MSCs-derived exosomes promote the proliferation of HCT116-CSCs.

### 3.3. BM-MSCs-Derived Exosomes Inhibit Apoptosis and Alter Cell Cycle of HCT116-CSCs

Flow cytometry analysis showed that the early apoptotic rate of HCT116-CSCs was significantly lower in the Exo group than in the PBS group and control group (*p* < 0.05), while the late apoptotic rate showed no significant difference among the groups (Figure 2D). This suggests that BM-MSCs-derived exosomes primarily inhibit early apoptosis in HCT116-CSCs. Furthermore, cell cycle analysis revealed a significant decrease in the percentage of cells in the G0/G1 phase and a concurrent increase in the percentage of cells in the S phase in the Exo group compared to the control groups (Figure 2E). These data indicate that BM-MSCs-derived exosomes may promote the proliferation of HCT116-CSCs by regulating cell cycle progression.

### 3.4. BM-MSCs-Derived Exosomes Promote HCT116-CSCs Migration and Invasion

The Transwell assay demonstrated that the number of HCT116-CSCs that migrated through the membrane without Matrigel was significantly higher in the Exo group than in the control groups (*p* < 0.01). Similarly, the numbers of cells that invaded through the Matrigel-coated membrane were also markedly increased in the Exo group (*p* < 0.01) (Figure 2F). These results indicate that BM-MSCs-derived exosomes significantly enhance the migratory and invasive capacities of HCT116-CSCs.

### 3.5. BM-MSCs-Derived Exosomes Promote the Tumorigenesis and Lung Metastasis of HCT116-CSCs *in Vivo*

**Figure 3:**
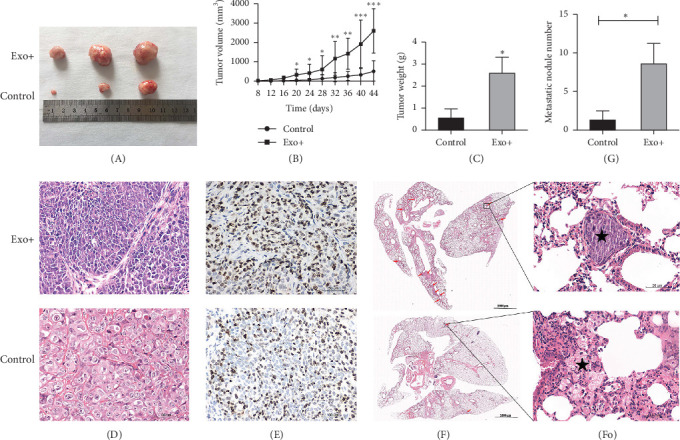
Rat BM-MSC-Exos promote the tumorigenesis and metastasis of HCT116-CSCs in vivo. (A) Representative images of xenograft tumors excised from nude mice 42 days postinjection. (B) The growth curve of xenograft tumors. *⁣*^*∗*^*p*  < 0.05, *⁣*^*∗∗*^*p*  < 0.01, *⁣*^*∗∗∗*^*p*  < 0.001 vs. the control group. (C) Weights of xenograft tumors. *⁣*^*∗*^*p*  < 0.05 vs. the control group. (D) H&E staining of xenograft tumors tissues. Scale bar = 50 μm. (E) Immunohistochemistry staining for Ki-67 in xenograft tumors. Scale bar = 50 μm. (F) H&E staining of lung tissues showing metastatic foci (indicated by arrows). Scale bar = 1000 μm. (Fo) Higher magnification of the boxed area in (F). Scale bar = 50 μm. (G) Quantification of metastatic lung nodules. *⁣*^*∗*^*p*  < 0.01 vs. the control group.

The in vivo tumorigenicity assay showed a comparable subcutaneous tumor formation rate (three out of five mice) in both the experimental and control groups. However, the tumors in the experimental group exhibited significantly enhanced growth capacity compared to those in the control group (Figure 3A). Tumor volumes, measured every 4 days from the third week onward, were significantly larger in the experimental group than in the control group (Figure 3B). At the endpoint (42 days postinjection), the excised tumors in the experimental group were significantly heavier than those in the control group (Figure 3C). H&E staining of lung tissues revealed the formation of metastatic nodules was significantly higher compared to the control group (Figure 3D, F, and G). Immunohistochemical staining of xenograft tumors showed a clear increase in Ki-67 expression in the experimental group, indicating enhanced proliferation (Figure 3E). These results demonstrate that BM-MSC-derived exosomes promote tumor growth and lung metastasis of HCT116-CSCs *in vivo*.

### 3.6. BM-MSCs-Derived Exosomes Promote HCT116-CSCs Invasion and Metastasis by Inducing Epithelial-Mesenchymal Transition (EMT)

**Figure 4:**
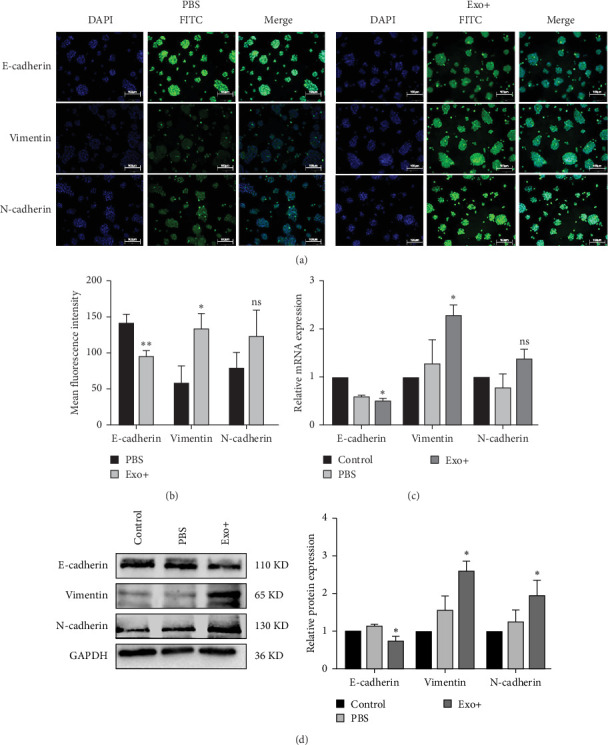
Rat BM-MSC-Exos induce EMT in HCT116-CSCs in vitro. (A) Immunofluorescence staining of E-cadherin, Vimentin, and N-cadherin in HCT116-CSCs. Scale bar = 50 μm. (B) Quantification of fluorescence intensity for E-cadherin, Vimentin, and N-cadherin. *⁣*^*∗*^*p*  < 0.05, *⁣*^*∗∗*^*p*  < 0.01 vs. the PBS group. (C) qPCR analysis of E-cadherin, Vimentin, and N-cadherin mRNA expression. *⁣*^*∗*^*p*  < 0.05 vs. the PBS group; ns, not significant. (D) Western blot analysis and quantification of EMT marker proteins. *⁣*^*∗*^*p*  < 0.05 vs. the PBS group.

To investigate the mechanism, we examined the expression of EMT markers. Immunofluorescence staining detected significantly lower expression of E-cadherin (*p* < 0.01) and higher expression of Vimentin (*p* < 0.05) in the Exo group compared to the PBS group (Figure 4A, B). qPCR analysis confirmed a significantly downregulation of E-cadherin mRNA (*p* < 0.05) and upregulation of Vimentin mRNA (*p* < 0.05) in the Exo group (Figure 4C). Western blot analysis further demonstrated a significantly decrease in E-cadherin protein expression and a significant increase in Vimentin and N-cadherin protein expression in the Exo group (Figure 4D). These in vitro findings indicate that BM-MSCs-derived exosomes induce EMT in HCT116-CSCs.

**Figure 5:**
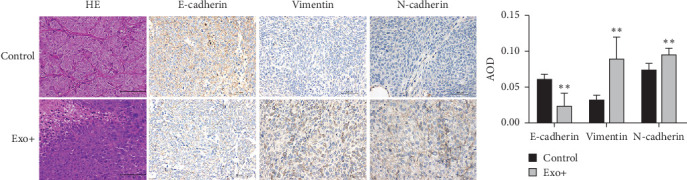
Immunohistochemical analysis of EMT markers in xenograft tumors. Scale bar = 50 μm. *⁣*^*∗∗*^*p*  < 0.01 vs. the control group.

Consistent with the in vitro results, immunohistochemical staining of xenograft tumors revealed lower expression of E-cadherin and higher expression of N-cadherin and Vimentin in the experimental group compared to the control group (Figure 5). This confirms that BM-MSC-derived exosomes promote the invasion and metastasis of HCT116-CSCs in vivo via induction of EMT.

## 4. Discussion

Our study demonstrates that exosomes derived from rat BM-MSCs promote malignant phenotypes in human CRC-CSCs, including enhanced proliferation, migration, invasion, and metastasis, coupled with inhibited apoptosis. These effects are mechanistically driven by the induction of EMT. These findings provide novel insights into how the TME facilitates CRC progression through vesicle-mediated intercellular communication.

The plasticity and aggressiveness of CSCs are strongly shaped by their niche within the TME [21]. MSCs are a crucial stromal component that can migrate to tumor sites and support tumorigenesis [22]. Our previous work established that rat BM-MSCs directly enhance the invasion and migration of human CRC-CSCs [14], but the mechanisms remained elusive. Given that MSCs primarily communicate via secreted factors, including exosomes [23, 24], we hypothesized that exosomes may serve as key mediators in this process.

In this study, we successfully isolated and characterized exosomes derived from rat BM-MSCs. Functional assays confirmed that these exosomes are potent enhancers of CRC-CSC aggressiveness, promoting proliferation, migration, invasion, and cell cycle progression in vitro while suppressing apoptosis. In vivo, these exosomes significantly promoted tumor growth and lung metastasis.

A central question pertained to the mechanism underlying these effects. Our data strongly indicate that activation of the EMT program serves as the pivotal mechanism. We consistently observed downregulation of the epithelial marker E-cadherin and upregulation of the mesenchymal markers Vimentin and N-cadherin following exosome treatment in both cultured cells and xenograft tumors. EMT is a well-established driver of metastasis, conferring upon cancer cells enhanced motility, invasiveness, and resistance to apoptosis [25]. Notably, the stemness properties of CSCs and EMT are closely interconnected; cells undergoing EMT often acquire stem-like characteristics, and CSCs frequently exhibit an EMT phenotype [26, 27]. This interplay establishes a self-reinforcing cycle that promotes tumor progression and therapeutic resistance.

The observed alterations in the cell cycle—a reduction in the G0/G1 phase and a corresponding accumulation in the S phase—further support a pro-proliferative activation state induced by the exosomes. This shift is mechanistically linked to EMT, as key signaling pathways driving EMT (e.g., Wnt/β-catenin, PI3K/Akt) are also potent regulators of cell cycle progression, known to inhibit G1 arrest and promote S-phase entry [28–30]. Therefore, the molecular cargo within BM-MSC-derived exosomes likely activates these common upstream pathways, orchestrating a coordinated cellular response that enhances both proliferative and metastatic potential, thereby expanding the expansion of the aggressive CSC population.

Our findings are consistent with the accumulating evidence that underscores stromal-derived exosomes as key regulators of the TME. For example, cancer-associated fibroblasts (CAFs) have been demonstrated to enhance stemness and induce EMT in CRC cells via exosomal transfer [31]. This study expands that concept by identifying BM-MSC-derived exosomes as another potent stromal contributor to CRC aggressiveness. The internalization of these exosomes by CSCs establishes a direct communication pathway, effectively reprograming the recipient cells toward a more malignant phenotype.

### 4.1. Limitations and Future Perspectives

Although our findings elucidate the role of BM-MSC-derived exosomes, several limitations should be acknowledged. First, using only one CSC-enriched cell line (HCT116) may not capture the full heterogeneity of CRC. Future studies should validate these findings using additional CRC lines or patient-derived organoids. Second, our cross-species model (rat-derived exosomes applied to human cells), while useful for mechanistic discovery, may introduce biological variability. Experiments using human BM-MSC-derived exosomes are necessary to confirm the clinical relevance of our results. Third, the specific molecular cargo within BM-MSC-exosomes (e.g., specific miRNAs, proteins, or lipids) responsible for inducing EMT remains unidentified. Proteomic and transcriptomic analyses of these exosomes are essential next steps to identify the functional mediators. Furthermore, the use of a single exosome dose, while effective, leaves room for optimization. Future studies examining a dose-dependent response will be essential. Last, the lack of a control group treated with exosomes from non-MSC sources (e.g., fibroblasts) precludes definitive conclusions regarding whether the observed effects are specific to BM-MSC-derived exosomes or representative of a more general exosome-mediated phenomenon. The inclusion of such a control group will be essential in future studies.

## 5. Conclusion

In conclusion, our data identify a novel mechanism within the CRC TME whereby exosomes derived from BM-MSCs drive cancer progression through the activation of EMT in CSCs. From a translational perspective, this study suggests that targeting this specific pathway—by inhibiting exosome biogenesis, secretion, uptake, or by neutralizing their key oncogenic cargo—may offer a promising therapeutic strategy to disrupt pro-tumorigenic intercellular crosstalk. Integrating such targeted interventions with conventional chemotherapy or immunotherapy could help suppress CSC-driven metastasis and overcome therapeutic resistance, ultimately improving clinical outcomes for patients with advanced CRC.

## Figures and Tables

**Table 1:** Sequences of primers used for qPCR assay.

Gene	Primer sequences
*E-cadherin*	Forward: 5′-GAACGCATTGCCACATACAC-3′; Reverse: 5′-GAGGATGGTGTAAGCGATGG-3′

*Vimentin*	Forward: 5′-GACGCCATCAACACCGAGTT-3′; Reverse: 5′-CTTTGTCGTTGGTTAGCTGGT-3′

*N-cadherin*	Forward: 5′-TGGACCATCACTCGGCTTA-3′; Reverse: 5′-ACACTGGCAAACCTTCACG-3′

*GAPDH*	Forward: 5′-GAAGGTGAAGGTCGGAGTC-3′; Reverse: 5′-GAAGATGGTGATGGGATTTC-3′

## Data Availability

The data that support the findings of this study are available from the corresponding author upon reasonable request.

## References

[1] Dekker E., Tanis P. J., Vleugels J. L. A., Kasi P. M., Wallace M. B. (2019). Colorectal Cancer. *The Lancet*.

[2] Bray F., Laversanne M., Sung H. (2024). Global Cancer Statistics 2022: GLOBOCAN Estimates of Incidence and Mortality Worldwide for 36 Cancers in 185 Countries. *CA: A Cancer Journal for Clinicians*.

[3] Bisht S., Nigam M., Kunjwal S. S., Sergey P., Mishra A. P., Sharifi-Rad J. (2022). Cancer Stem Cells: From an Insight Into the Basics to Recent Advances and Therapeutic Targeting. *Stem Cells International*.

[4] Batlle E., Clevers H. (2017). Cancer Stem Cells Revisited. *Nature Medicine*.

[5] Zhou H., Tan L., Liu B., Guan X.-Y. (2023). Cancer Stem Cells: Recent Insights and Therapies. *Biochemical Pharmacology*.

[6] Das P. K., Islam F., Lam A. K. (2020). The Roles of Cancer Stem Cells and Therapy Resistance in Colorectal Carcinoma. *Cells*.

[7] Hervieu C., Christou N., Battu S., Mathonnet M. (2021). The Role of Cancer Stem Cells in Colorectal Cancer: From the Basics to Novel Clinical Trials. *Cancers*.

[8] De Angelis M. L., Francescangeli F., Zeuner A., Baiocchi M. (2021). Colorectal Cancer Stem Cells: An Overview of Evolving Methods and Concepts. *Cancers*.

[9] Gupta R., Bhatt L. K., Johnston T. P., Prabhavalkar K. S. (2019). Colon Cancer Stem Cells: Potential Target for the Treatment of Colorectal Cancer. *Cancer Biology & Therapy*.

[10] Munro M. J., Wickremesekera S. K., Peng L., Tan S. T., Itinteang T. (2018). Cancer Stem Cells in Colorectal Cancer: A Review. *Journal of Clinical Pathology*.

[11] Huang T. X., Guan X. Y., Fu L. (2019). Therapeutic Targeting of the Crosstalk Between Cancer-Associated Fibroblasts and Cancer Stem Cells. *American Journal of Cancer Research*.

[12] Zhou J., Shi Y. (2023). Mesenchymal Stem/Stromal Cells (MSCs): Origin, Immune Regulation, and Clinical Applications. *Cellular & Molecular Immunology*.

[13] Ridge S. M., Sullivan F. J., Glynn S. A. (2017). Mesenchymal Stem Cells: Key Players in Cancer Progression. *Molecular Cancer*.

[14] Zou W., Zhao J., Li Y. (2020). Rat Bone Marrow-Derived Mesenchymal Stem Cells Promote the Migration and Invasion of Colorectal Cancer Stem Cells. *Onco Targets and Therapy*.

[15] Zou W., Zhang Y., Bai G. (2022). siRNA-Induced CD44 Knockdown Suppresses the Proliferation and Invasion of Colorectal Cancer Stem Cells Through Inhibiting Epithelial-Mesenchymal Transition. *Journal of Cellular and Molecular Medicine*.

[16] Rezaie J., Feghhi M., Etemadi T. (2022). A Review on Exosomes Application in Clinical Trials: Perspective, Questions, and Challenges. *Cell Communication and Signaling*.

[17] Perocheau D., Touramanidou L., Gurung S., Gissen P., Baruteau J. (2021). Clinical Applications for Exosomes: Are We There yet?. *British Journal of Pharmacology*.

[18] Kou M., Huang L., Yang J. (2022). Mesenchymal Stem Cell-Derived Extracellular Vesicles for Immunomodulation and Regeneration: A Next Generation Therapeutic Tool?. *Cell Death & Disease*.

[19] Guo G., Tan Z., Liu Y., Shi F., She J. (2022). The Therapeutic Potential of Stem Cell-Derived Exosomes in the Ulcerative Colitis and Colorectal Cancer. *Stem Cell Research & Therapy*.

[20] Yang J., Zhang L. (2022). The Roles and Therapeutic Approaches of MSC-Derived Exosomes in Colorectal Cancer. *Clinical & Translational Oncology*.

[21] Bayik D., Lathia J. D. (2021). Cancer Stem Cell–Immune Cell Crosstalk in Tumour Progression. *Nature Reviews Cancer*.

[22] Zhang L. N., Zhang D. D., Yang L. (2021). Roles of Cell Fusion Between Mesenchymal Stromal/Stem Cells and malignant Cells in Tumor Growth and Metastasis. *The FEBS Journal*.

[23] Whiteside T. L. (2018). Exosome and Mesenchymal Stem Cell Cross-Talk in the Tumour Microenvironment. *Seminars in Immunology*.

[24] Weng Z. J., Zhang B., Wu C. Z. (2021). Therapeutic Roles of Mesenchymal Stem Cell-Derived Extracellular Vesicles in Cancer. *Journal of Hematology & Oncology*.

[25] Glaviano A., Lau H. S.-H., Carter L. M. (2025). Harnessing the Tumor Microenvironment: Targeted Cancer Therapies Through Modulation of Epithelial-Mesenchymal Transition. *Journal of Hematology & Oncology*.

[26] Olivares-Urbano M. A., Griñán-Lisón C., Marchal J. A., Núñez M. I. (2020). CSC Radioresistance: A Therapeutic Challenge to Improve Radiotherapy Effectiveness in Cancer. *Cells*.

[27] Shibue T., Weinberg R. A. (2017). EMT, CSCs, and Drug Resistance: The Mechanistic Link and Clinical Implications. *Nature Reviews Clinical Oncology*.

[28] Tanabe S., Quader S., Cabral H., Ono R. (2020). Interplay of EMT and CSC in Cancer and the Potential Therapeutic Strategies. *Frontiers in Pharmacology*.

[29] Zhao Z. Q., Wu X. J., Cheng Y. H. (2021). TROAP Regulates Cell Cycle and Promotes Tumor Progression Through Wnt/β-Catenin Signaling Pathway in Glioma Cells. *CNS Neuroscience & Therapeutics*.

[30] Wang Z. X. (2021). Regulation of Cell Cycle Progression by Growth Factor-Induced Cell Signaling. *Cells*.

[31] Hu J. L., Wang W., Lan X. L. (2019). CAFs Secreted Exosomes Promote Metastasis and Chemotherapy Resistance by Enhancing Cell Stemness and Epithelial-Mesenchymal Transition in Colorectal Cancer. *Molecular Cancer*.

